# The role of the anterior insula during targeted helping behavior in male rats

**DOI:** 10.1038/s41598-022-07365-3

**Published:** 2022-02-28

**Authors:** Stewart S. Cox, Angela M. Kearns, Samuel K. Woods, Brogan J. Brown, Samantha J. Brown, Carmela M. Reichel

**Affiliations:** Medical University of South Carolina, 173 Ashley Avenue, Basic Science Building Suite 416a, Charleston, SC 29425 USA

**Keywords:** Social behaviour, Reward

## Abstract

Empathy, the understanding of the emotional state of others, can be examined across species using the Perception Action Model, where shared affect promotes an action by “Observers” to aid a distressed “Target”. The anterior insula (AI) has garnered interest in empathic behavior due to its role integrating sensory and emotional information of self and other. In the following studies, the AI was inhibited pharmacologically and chemogenetically during targeted helping. We demonstrate the insula is active during, and is necessary for the maintenance of, targeted helping. Analysis of ultrasonic vocalizations revealed distress calls from Targets increased when Observers’ helping was attenuated due to insula inhibition. Targets’ elevated distress was directly correlated to Observers’ diminished helping behavior, suggesting emotional transfer between Observer and Target is blunted following Observer AI inhibition. Finally, the AI may selectively blunt targeted helping, as social exploration did not change in a social reward place conditioning task. These studies help further establish the anterior insula as a critical node in the empathic brain during targeted helping, even in the absence of direct social contact.

## Introduction

Empathy is the capacity to share the feelings of another and generate an appropriate response to those shared feelings^1–4^. It is a multidimensional concept that shapes social behaviors and allows for the formation and maintenance of interpersonal relationships critical for social cohesion^5–8^. Further, it is becoming more apparent that empathy is dysregulated in numerous DSM-V-defined psychiatric disorders. Patients diagnosed with Substance Use Disorder^9–11^, Major Depressive Disorder^12–14^, and Autism Spectrum Disorder and alexithymia^15–17^, to name a few, have blunted empathic processes, which have been shown to be directly correlated with the severity of disease^12^ and reduced quality of life^18^. The role empathy plays in social behaviors and the prevalence of its dysregulation in psychiatric disorders make its behavioral and neurobiological understanding paramount.

One theory used to understand the complexity of empathy is the perception action model (PAM). According to this theory, an “Observer” must attend to a conspecific’s (labeled the “Target”) distress, thereby generating a shared affective state between them^19–21^. The Observer must regulate their emotional responsivity to effectively perform a behavior that will eliminate the distress of the Target and, by extension, their own. This phenomenon is the basis of more complex empathic behaviors seen across numerous mammalian species, from rodents to humans^20,22,23^. To best understand empathic processes is through translationally relevant rodent models. Several rodent models are used to understand different aspects of empathy, including emotional contagion, observational fear learning, and targeted helping (reviewed in^21^). Our lab has created and validated a targeted helping model that demonstrates rats release a distressed conspecific independent of direct contact, suggesting the rewarding effects of social interaction is not necessary to see prosocial behaviors and the helping behavior observed may be primarily driven by the desire to reduce the distress of another^24^. We further validated that the behavior in the task was specific to the presence of a distressed conspecific, was persistent, and subject to effort, suggesting the model appropriately adheres to the PAM^24^.

Although there is still a dearth of understanding in the underlying neurobiology of empathic behaviors, one region that has gained significant interest is the anterior insula (AI). The insula is an anatomically and functionally heterogeneous region uniquely positioned to be a primary node in multiple complex functions including sensory processing, risk prediction, emotional representation, and decision-making^25–27^. Moreover, the AI has been implicated in integrating interoceptive and affective information^28,29^, functions necessary for prosocial and empathic behaviors^6,30^. In animal studies, the insula has been implicated in social approach and avoidance behaviors under certain social contexts^31^. Further, inactivation of the insula reverses hyperalgesia induced by emotional contagion of pain^32^, while chemogenetic activation of the AI restores a heroin-induced decrease in targeted helping^33^. These data seem to strongly correlate to clinical findings, as functional neuroimaging studies demonstrate an overlapping activity pattern in the AI when directly receiving, or vicariously viewing another receive a painful stimulus^34–36^. Additionally, patients with epilepsy having undergone insular resection had impairments in the ability to recognize others’ facial expressions^37,38^. Aberrant insular activity also plays a role in the dysregulation of empathic behaviors seen in patients with Autism Spectrum Disorder^39^. Combined, these studies suggest the AI has the potential to be a translationally relevant target for understanding empathy. However, it is uncertain if the AI plays a role in an empathic behavior in which social interaction does not act as an inherent reward following the completion of the task. To answer this question, we used our lab’s social contact-independent model of targeted helping^24^ to elucidate the role of the AI during empathic behavior. Using this model, we have shown rats readily aid a distressed conspecific in the absence of the opportunity for social contact, and previous experience in the distressing condition significantly reduces the latency rats take to aid another, as predicted in the PAM^24^. In this study, we pharmacologically (Experiment 1) and chemogenetically (Experiment 2) inhibited the AI of observers during targeted helping. We demonstrate that the insula is activated during targeted helping, while its inhibition blunts this behavior and the associated change in helping directly affects the distress of the Target, as measured by ultrasonic vocalizations. Further, AI inhibition was selective for targeted helping and not prosocial behaviors per se, as social exploration and interaction with an unfamiliar rat was unchanged. These results help further corroborate the PAM in rats and the role of the AI as a key substrate in empathic behaviors independent of social contact.

## Results

**Figure 1:**
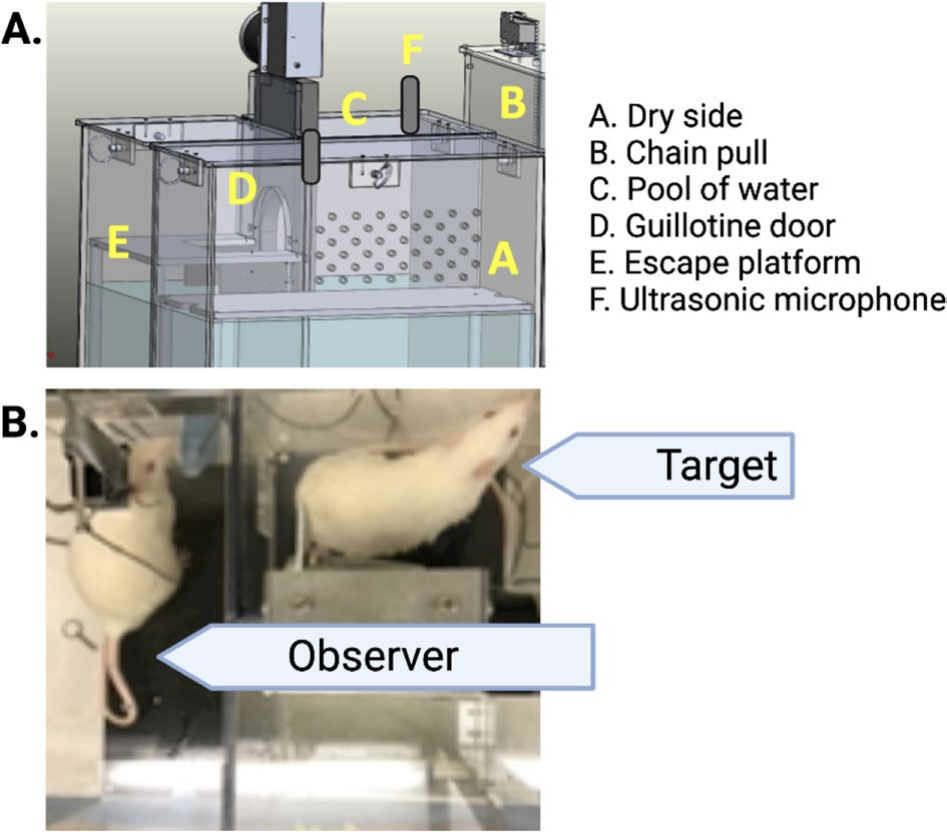
The insula is active during targeted helping. (**A**) Depiction of the apparatus used during the social contact-independent targeted helping task. Observers pull a chain that opens an automated guillotine door, allowing the Target to escape the distressing context into a separate dry platform. Ultrasonic vocalizations were recorded in some of the following experiments using two high quality condenser microphones attached to the top of the dry and water compartments. (**B**) This photo depicts an Observer in the dry side of the apparatus and a Target on the escape platform after being released from the pool of water.

Figure 1A shows configuration of our chamber in which targeted helping is evaluated. Figure 1B depicts an observer rat in the dry compartment and a target rat on the escape platform.

### Experiment 1: pharmacological inhibition of the AI

**Figure 2:**
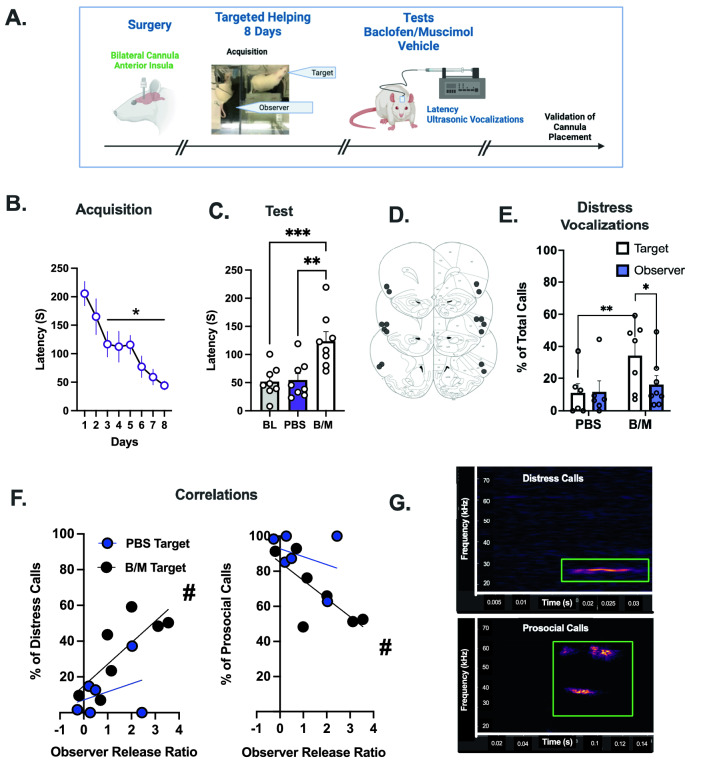
Pharmacological Inhibition of the AI Attenuates Targeted Helping. (**A**) A timeline for Experiment 2. Direct pharmacological inhibition of the anterior insula (AI) via bilateral indwelling cannulae was performed to determine if AI activity is necessary for social contact-independent targeted helping. Following surgeries, Observers performed 8 days of acquisition in the targeted helping task. (**B**) The latency for Observers to release the Target significantly decreased across time, with the latencies on days 3–8 significantly faster compared to day 1, indicating Observers learned to release their conspecific. (**C**) On test days 9–10, rats either received direct micro infusions of baclofen/muscimol (B/M) or phosphate buffered saline (PBS) into the AI. Rats that received B/M demonstrated a significantly potentiated change in latency compared to the PBS group and to baseline (BL; average of the last 2 days of acquisition). (**D**) A representative image of the cannulae placement of each rat bilaterally. (**E**) Ultrasonic vocalizations (USV) were recorded on test days, and distress calls were analyzed as a proportion of total calls made during the task. Targets made significantly more USV within the distress frequency range on tests days when their respective Observer received B/M infusions compared to PBS. (**F**) To understand the relationship between the blunted helping behavior and enhanced distress calls, a Pearson correlation was performed between Observers’ release ratio ($\frac{{Acq}_{Test}-{Acq}_{BL}}{{Acq}_{BL}}$) and the percent of distress (left) and prosocial (right) calls made by their respective Targets. A significant correlation was found on days when Observers received B/M infusion, but not PBS. (**G**) Representative images of USVs that fall within the distress frequency range (18–35 kHz) and prosocial range (> 35 kHz) are shown here. *significant difference from day 1, p < 0.05. **significant difference from PBS, p < 0.005. ***significant difference from BL, p < 0.005. **#** Significant correlation, p < 0.05.

The first experiment temporarily inactivated the AI with micro injection of a baclofen/muscimol (BM) cocktail. The experimental timeline (Fig. 2A) shows that Observers (n = 8) underwent stereotaxic surgery in which indwelling cannula were implanted bilaterally into the AI. During acquisition, there was a main effect of time [Fig. 2B, F (7,49) = 6.683, p < 0.0001], and post hoc analysis showed reduced latencies to release Targets on days 3–8 compared to day 1 (p < 0.05). On test days, Observers were directly infused with either a B/M cocktail or a phosphate-buffered saline (PBS) control bilaterally into the AI and chain pull latencies were recorded. Rats tested on both conditions and treatments were counter balanced such that half the rats tested on B/M first and half on PBS first. A baseline (BL) was calculated by averaging the last 2 days of acquisition (days 7–8). A repeated measures (RM) one-way ANOVA showed a main effect of infusion [F (2,14) = 12.36, p = 0.0008]. Post hoc analysis showed longer latencies when Observers received B/M infusions relative to both BL (p = 0.0018) and PBS (p = 0.0018, Fig. 2C). Canula placements are schematically depicted in Fig. 2D.

During the PBS and B/M sessions, ultrasonic vocalizations (USVs) were recorded from the targets and observers. Data were analysed with a 2-way mixed effects ANOVA, there were no main effects or interactions to indicate differences in the total amount of calls during the full 300 s sessions or in Targets and Observers (Supplemental Fig. 1). Calls were divided into two main USVs categorizations based on kHz: distress (between 18–35 kHz) and prosocial/appetitive (> 35 kHz) calls^25,26^. Calls within the distress frequency range (18–35 kHz) were analyzed with a 2-way mixed effects ANOVA. Figure 2E depicts the proportion of distress calls made by Targets and Observers on PBS and B/M trials. There was a main effect of test [F (1, 9) = 22.26, p = 0.0011)], as well as a test x group interaction [F (1,9) = 7.724, p = 0.0214)]. Post hoc analysis showed Target rats of Observer partners that received B/M infusions had a significantly larger proportion of their total calls fall within the distress range compared to Targets on days where their corresponding Observers received PBS control (p = 0.0012) infusions and relative to their Observer (p = 0.049). To determine the relationship between Observers’ helping behavior and Targets’ proportion of distress calls and prosocial calls, we first calculated a Release Ratio with the following formula: ($Release Ratio= \frac{{Acq}_{Test}-{Acq}_{BL}}{{Acq}_{BL}}$) as a ratio of the change in chain pull latency from baseline to test day (either PBS or B/M infusion). Pearson r correlations were performed between the Observers’ Release Ratio and the proportion of distress or prosocial calls made by Targets. For distress calls, the correlations in PBS trials were not significant (r = 0.346, p = 0.25), but there was a positive correlation on B/M trials between the Observers’ release ratio and the proportion of the Targets’ calls in the distress range (r = 0.7732, p = 0.0207). The inverse was found for the proportion of prosocial calls. PBS trials did not correlate (r = 0.346, p = 0.25), whereas prosocial calls were negatively correlated with release latency (r = 0.735, p = 0.03) (Fig. 2F). Figure 2G depicts a representative sample image of a distress and prosocial calls with the 18–35 kHz range or over 35 kHz range, respectively.

### Experiment 2: chemogenetic inhibition of the AI

**Figure 3:**
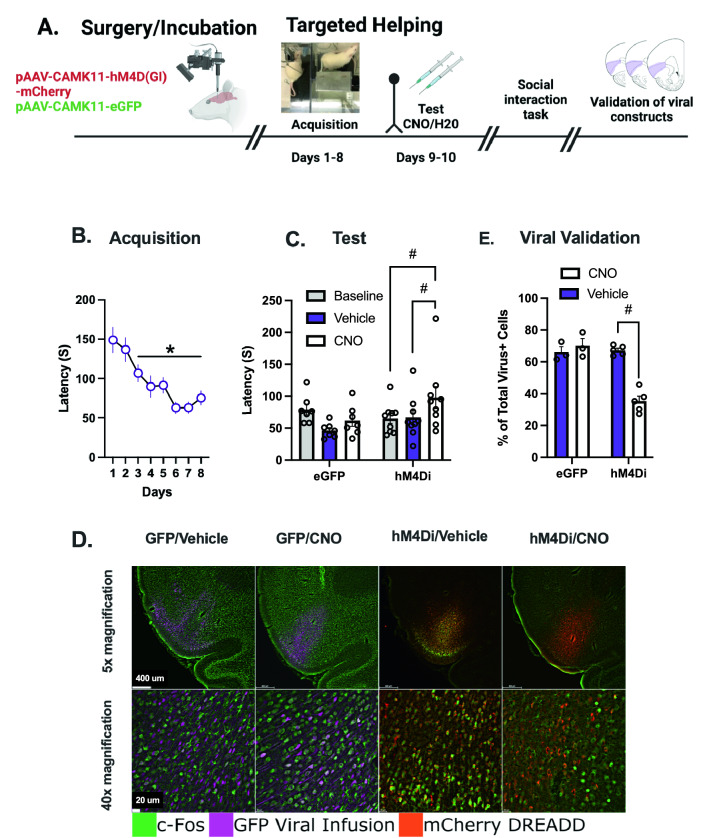
Experiment 3, Chemogenetic Inhibition of the AI Attenuates Targeted Helping. (**A**) Experimental 3 timeline. Male Sprague Dawley Observer rats underwent stereotax surgeries and received micro infusions of either the control virus (AAV8-CaMKIIα-EGFP, “EGFP”, n = 8) or the inhibitory DREADD virus (AAV8-CaMKIIα-hM4D(Gi), “hM4Di”, n = 10). After 3 weeks of recovery and viral incubation, rats went through the targeted helping task for 8 days. On the final 2 days of targeted helping, Observers received either clozapine-N-oxide (CNO) or water injections (i.p.) in a within-subjects experimental paradigm. Next, rats underwent a social reward place conditioning task (shown in Fig. 4). On the day animals were sacrificed, Observers were injected with either CNO or water and again performed the targeted helping task. (**B**) During the targeted helping task, Observers learned to release a distressed conspecific faster on days 3–8 compared to day 1. (**C)** On test days, Observers infused with the Gi DREADD injected with CNO had latencies significantly higher than their baseline (BL, days 7–8) and vehicle. There were no differences in eGFP viral control animals in response to CNO or vehicle. (**D**) Representative images of the AI in the viral control (EGFP) and hM4Di groups with either vehicle or CNO infusions. (**E**) 90 min following targeted helping, the animals were sacrificed to stain for *c-fos* in the AI to confirm both placement and activity of the inhibitory DREADDs by quantifying Fos + /Virus + cells as a percentage of total Virus + cells. Rats having received the Gi DREADD infusion and CNO injections 30 min prior to behavior had significantly fewer Fos + /Virus + cells compared to all other animals, indicating the DREADD construct was activated exclusively following CNO injection. *Significant difference from day 1, p < 0.05. **#**Significant difference from Veh and/or BL, p < 0.05

Having demonstrated that pharmacological inactivation of the AI interfered with targeting helping, we sought to replicate this effect through Chemogenetic inhibition. The schematic for Experiment 3 is depicted in Fig. 3A. Observer rats had either a viral vector control (EGFP) or an inhibitory DREADD (hM4Di) bilaterally infused into the AI. Figure 3B shows chain pull latency was significantly reduced on days 3–8 compared to day 1 [F (7, 117) = 8.14, p < 0.001 and post hoc P < 0.05) in a mixed effects model. On test days rats received intraperitoneal (i.p.) injections of either clozapine-N-oxide (CNO, n = 9) or a vehicle injection (n = 7). There was a main effect of injection [F (2,28) = 4.614, p = 0.0185)] and an injection x virus interaction [F (2,28) = 5.453, p = 0.01]. Post hoc analysis confirmed CNO increased release latency in hM4Di rats compared to both BL acquisition (p = 0.0107) and vehicle injections (p = 0.0111, Fig. 3C).

Viral placement and efficacy were confirmed following behavior in the targeted helping task by staining for *c-fos* and quantifying Fos + /virus + cells within the AI as a percentage of total virus + cells (representative images depicted in Fig. 3D). Refer to Supplemental Table 1 for mean count values. A 2-way ANOVA demonstrated main effects of viral group [F (1, 12) = 29.95, p = 0.0001)] and injection [F (1, 12) = 20.50, p = 0.0007)], and a significant viral group x injection interaction [F (1, 12) = 33.99, p < 0.0001)]. Post hoc analysis showed that hm4Di + CNO rats had less Fos + /virus + cells compared to all other groups (Fig. 3E).

**Figure 4:**
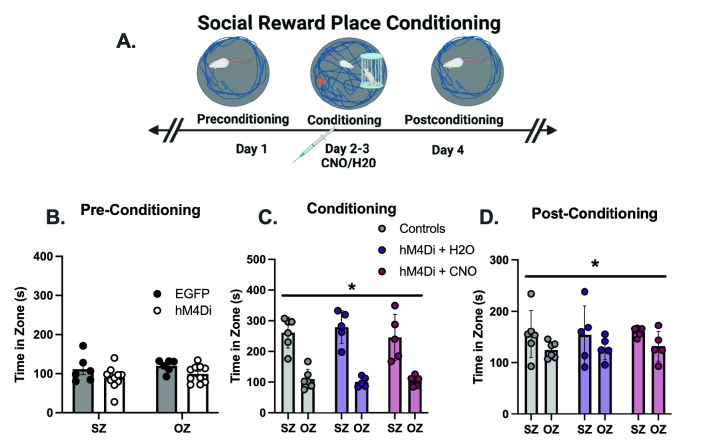
Social Interaction Does Not Differ Following Insula Inhibition. (**A**) Rats underwent a social reward place conditioning task. On preconditioning day 1, rats were placed in the open field and given 10 min to explore the environment. On conditioning days 2–4, Observers were injected with water or CNO 30 min prior to the task in a between subject’s design and given the opportunity to explore an unfamiliar rat and an unfamiliar object simultaneously. Finally, on day 5, rats were returned to the empty open field for a post-conditioning assessment. Time spent in the social zone (SZ) and the object zone (OZ) were recorded. (**B**) Rats did not exhibit a side bias within the open field, equally preferring the SZ and OZ on pre-conditioning day. (**C)** On conditioning days, a main effect of zone was observed in which all groups preferred the SZ over the OZ. (**D**) During post-conditioning day 5, a main effect of side was again observed, in which the time spent in the SZ was greater than the OZ. However, all groups equally preferred the SZ over the OZ. *Significant effect of zone, p < 0.05.

These rats also underwent a social reward place preference task (see Fig. 4A for timeline) to determine if AI inhibition would decrease contact with a social partner. No behavioral differences were found in control rats (EGFP) between water and CNO injections, so these data were combined for analysis. The cumulative duration of EGFP and hM4Di groups did not differ during preconditioning in the time spent in the social zone (SZ) or object zone (OZ) (Fig. 4B). On conditioning days 2–4, a main effect of zone was seen [F (1,13) = 16.94, p < 0.0001)], with all groups spending significantly more time in the SZ compared to the OZ (Fig. 4C). However, groups did not differ from one another in their time spent within the SZ. In addition, no other indicators of social interaction, like nose-nose contact between animals and time spent cage climbing, nor other indicators of novel object exploration (object climbing) differed significantly between groups (Supplemental Fig. 2A–C). On postconditioning day 4, a main effect of zone was again found [F (1,13) = 5.028, p = 0.0430)]. No main effect of group was seen, indicating all groups equally retained a preference for the SZ (Fig. 4C). No difference in locomotion was seen between groups on any day (Supplemental Fig. 2D).

## Discussion

The anterior insula (AI) has been implicated as a critical neural substrate for numerous prosocial behaviors in both rodents^31–33^ and humans^34–38^, potentially making it a translationally relevant region controlling empathic behavior. However, until now it was unknown if the role of the AI in rats was related to the rewarding effects of social contact or the process of aiding a conspecific. In the series of studies outlined in this manuscript, we elucidate the role of the anterior insula (AI) during social contact-independent targeted helping^24^. Overall, we report inhibition of the AI significantly blunts release behavior during the social contact-independent targeted helping task. Further, the change in Observers’ helping behavior due to AI inhibition correlates to an increase in distress of the Target as measured by ultrasonic vocalizations. Further, the modulation of prosocial behavior due to blunted AI activity seems to be specific to targeted helping, as it did not affect behavior in the social reward place preference task. These findings provide further insight into the neural basis of social contact-independent helping behaviors and is consistent with both preclinical^32^ and clinical research^34–39^ that point to the insula as a primary node in the empathic brain.

Inhibition of the AI through both pharmacological (B/M cocktail) and chemogenetic (hM4Di) methods, significantly attenuated empathic behavior, as measured by a potentiation of chain pull latency during targeted helping. We report a novel finding in the relationship between the change in Observers’ release behavior and the Targets’ distress as assessed by ultrasonic vocalizations. In the trials where release latency was potentiated due to AI inhibition, the proportion of Targets’ USV that were within the distress frequency range increased significantly (Fig. 2E). Further, the Observers’ release ratio correlated with their Targets’ proportion of distress and prosocial USVs (Fig. 2F). This finding may reflect a time function between release ratio and proportion of calls, but the correlations occurred only on the B/M sessions and there were no differences in total call counts during the entirety of session, nor was there a correlation between call count and release ratio (Supplemental Fig. 1). As such, these data are more indicative of an association between the action of the Observers (or lack thereof when AI activity was blunted) and the affective states of their respective Targets, as evaluated by USV frequency^40,41^. These results also suggest the AI modulates affective transfer and/or shared affect between the two animals, as described in the perception action model (PAM) of empathy. Normally, emotional transfer of the Target to the Observer would drive behavior to reduce the distress of the Targets (and, by extension, the Observer). However, time to release is potentiated even in the context of an increasingly distressed conspecific, as indicated by increased distress calls, when AI activity is blunted. The inhibition of AI activity therefore likely diminishes the emotional salience or valence of the distress of the conspecific^25–27^, potentiating the time to release them from the water.

Although we observed an attenuation of helping behavior following AI inhibition, it did not affect social or novel object exploration or place preference (Fig. 4). Prior research reports that the insula is directly involved in object recognition memory^42^, and there is a large dopamine surge within the insula during the exploration of a novel object^43^. While the AI may modulate novel object exploration, its relatively low salience compared to exploration of a conspecific may have drowned out any effects during testing. AI inhibition also had no impact on social interaction with a conspecific in this task, which is antithetical to reports showing inhibition of the insula disrupts social exploration of an unfamiliar animal^44^, while chemogenetic activation of the AI subsequently restored heroin-induced deficits in prosocial behaviors^39^. However, it may corroborate findings in other studies showing the AI may modulate social exploration in a context-dependent manner. Instead of social interaction, per se, social cognition under circumstances of varying emotional or reward contexts may be controlled via the insula^45^. Therefore, it is possible that the DREADD-induced insular inhibition may not have been robust enough to attenuate social exploration to a significant degree, but adequate to see changes in targeted helping because inhibitory DREADDs have been shown to suppress approximately 50–60% of the neural activity of affected cells following CNO injections (Fig. 3B)^39,45^. Alternatively, it may be prosocial behaviors that are modulated via insular activity are not tested in the reward place conditioning task.

The AI acts as an “emotional integration hub”, receiving external sensory and interoceptive inputs from the thalamus, sensory cortex, and olfactory bulb, while also having strong connectivity with the limbic system and cortical regions^27,46^. This unique connectivity permits the AI to detect salience and assess valence of both self and other, and generate an appropriate prosocial behavior^27,45^. Therefore, it is likely the AI works within a circuit of neural substrates necessary to generate targeted helping outcomes via emotional transfer or shared affect. Alternative functions of the AI may have also contributed to the attenuation of targeted helping. For example, a primary cortical output of the AI is the premotor cortex, which may alter motor planning or activity^46,47^. Enthusiasm for this account is negated by the lack of motor activity changes during the social reward place conditioning (Supplemental Fig. 2D). The insula is also implicated in learning and memory, which may blunt operant recall during test^48^. However, inhibition of the AI did not blunt place memory on postconditioning day 4 of the social reward place conditioning task (Fig. 4D). Overall, AI inhibition disrupts targeted helping broadly, but further studies are necessary to parse apart its specific role within targeted helping.

There are several methodological considerations to address in this series of experiments. First, intercranial B/M may have spread to areas beyond the AI such as the orbital frontal cortex. Therefore, behavioral changes measured may have been impacted by inhibition of anatomically adjacent regions. This is negated by replication of AI inhibition with hM4Di in which we validated that viral spread was restricted to the AI. Third, 10 mg/kg CNO is higher than some suggest as a recommended range^49^, but our lab has consistently seen behavioral changes with this dose in the past^50,51^. Further, we used an EGFP control virus lacking the DREADD coding sequence to ensure that CNO or the reverse metabolite clozapine did not produce any observable behavioral effects as has previously been reported^52^. We also had a vehicle control group to confirm the inhibitory DREADD alone did not alter neural activity. Finally, this data set is limited to male subjects. The extent to which AI inhibition affects targeted helping in females is unknown. The few studies that do test sex differences show conflicting results; while some find empathic behaviors are greater in females^53^, others show no sex differences^54,55^. Fewer still look at underlying neurobiological differences between sex during empathic behaviors. It is therefore imperative to determine if the role of the AI is similar in female as male rats.

In conclusion, our results have established the importance of the anterior insula in targeted helping by showing, in two separate studies, its activity is necessary for release of a distressed conspecific independent of social contact. The perception action model (PAM) of empathy, as measured by our targeted helping task, incorporates several behavioral components, such as learning and memory and affective transfer. Although we did replicate our findings showing targeted helping is attenuated following AI inhibition, additional behavioral experiments could provide more understanding about the specific components of empathy dysregulated by AI inhibition. We also demonstrated the behavioral change was specific to empathic dysregulation, as social and novel object exploration was unchanged by insula inhibition. Overall, these studies point to the insula as a promising translational node for empathic processes that can help us understand a complex, requisite social behavior.

## Materials and methods

See supplemental methods for complete statistical and methodological information.

### Ethics statement

All experimental procedures were conducted in accordance with the “Guide for the Care and Use of Laboratory Rats” (Institute of Laboratory Animal Resources on Life Sciences, National Research Council) and approved by the IACUC of the Medical University of South Carolina (IACUC-2021-00551 and -00451) and completed with ARRIVE guidelines. All procedures and methods were also performed under the relevant guidelines and regulations.

### Animals

Male Sprague Dawley rats weighing 250–275 g were pair-housed on a 12-h reversed light cycle (lights on at 1800). Animals were given food and water ad libitum until behavioral testing, when they were then switched to a daily stable intake (20 g) of rat chow (Harlan). Following arrival, rats were given at least 5 days to acclimate to their cage mate. Afterward, one rat was randomly selected to be the “Observer” and the other the “Target”. Animals were handled and weighed for 2 days, 5 min/day before the behavioral assessment. For all behavioral evaluations, rats were transported to the experiment room and left undisturbed for 5 min. The tasks were performed in a sound-attenuated room with the lights off except for a single lamp used for the experimenter to view the test.

### General surgical procedures

Observer rats were anesthetized using isoflurane vaporized for inhalation (4–5% for induction in a chamber, 2–3% through a nose cone for preparation and 1–3% for surgical anesthesia maintenance). In all experiments, the AI was targeted using the following coordinates relative to the skull and bregma: + 3 mm anteroposterior, ± 4 mm mediolateral, and -4 mm (guide cannula) or -5 mm (glass micropipette) dorsoventral according to a stereotaxic atlas^56^. Details regarding surgeries for Experiments 2–3 are located in the supplemental materials. Following all surgeries, ketorolac (2.0 mg/kg, IP; Sigma Chemical, St. Louis, MO, USA) and cefazolin (0.2 g/kg, Patterson Veterinary, Saint Paul, MN, USA) were given as an analgesic and antibiotic, respectively, and rats were given at least 5 days to recover before any behavior assessment began.

### Social contact-independent targeted helping

Social contact-independent helping behavior was evaluated using a custom (Med Associates; Fairfax, VT, USA) operant box with three chambers, as described previously^24^. Briefly, Targets were placed in 100 mm of water in the ‘wet’ compartment, while Observers were placed on a dry platform with access to a chain that, when pulled, opened an automated door and released the Target into a dry compartment separate from the Observer, preventing any opportunity for social interaction after the chain pull. Latency to chain pull was taken as an index of helping behavior. Trials (2 per day) were conducted daily spaced 1.5 h apart from one another during the rats’ dark cycle and lasted a total of 300 s (5 min) regardless of the chain pull latency to reduce the likelihood that removal from the apparatus was a motivating factor for the behavior. If the Observer did not pull the chain within the allotted time, the experimenter ended the trial and released the Target followed by the Observer.

### Social reward place conditioning

To assess whether the changes seen during targeted helping impacted other prosocial components, rats in Experiment 3 underwent a social reward place conditioning task (Fig. 4). This task occurred in a round open field (125-cm diameter), and behavior was recorded with EthoVision tracking software in 10-min sessions. Additional information is located in the supplemental methods section.

### Immunohistochemistry (IHC)

Subjects were sacrificed and perfused approximately 90 min following behavior and brains were processed for cannula placement (Expt. 1), and chemogenetic efficacy (Expt. 2). In all cases, rats were anesthetized with Equithesin and then transcardially perfused with 150–200 mL cold 0.9% saline followed by 400–500 mL of 10% buffered formalin. Brains were removed and postfixed in 10% formalin for 24 h, submerged in 20% sucrose/0.1% sodium azide solution for 48 h, and then sectioned into 50-µm tissue sections.

#### Validation of DREADD Function

50-µm tissue slices were permeabilized and blocked in 2% normal goat serum (NGS) and 2% Triton X-100 in PBS and were incubated in a rabbit anti-Fos primary (Millipore; 1:1000) overnight at 4 °C primary with 2% normal goat serum. Tissue was then incubated at room temperature for 5 h while protected from light in goat anti-rabbit Alexa 594 (Abcam,1:1000; RRID#2734147) in the rats infused with the control AAV8-CaMKIIα-enhanced green fluorescent protein (EGFP) viral construct (Addgene, Watertown, MA), or and goat anti-rabbit Alexa 488 (Invitrogen,1:1000; RRID#2633280) in those infused with the AAV8-CaMKIIα-hM4D(Gi)-mCherry inhibitory DREADD (hM4Di, Addgene). Slices were mounted and cover slipped with Prolong Gold, and representative images of the insula were taken at 10 × magnification using a Nikon fluorescent microscope. Surgical placement was determined by visualizing the spread of the viral fluorophore and comparing it to the corresponding coronal section of the insula from the stereotaxic atlas^56^. Subjects were eliminated from the final dataset if no expression was visible in the cell body region or if there was substantial spread into adjacent regions. CNO-mediated insula activity change was determined by counting the overlap of Fos + cells and either mCherry (hM4Di) or EGFP (control viral vector) under experimenter-blind conditions. All images were quantified using ImageJ software (NIH).

### Ultrasonic vocalizations

Ultrasonic vocalizations (USVs) were recorded in Experiment 1 by fastening two high-quality condenser microphones (Avisoft Bioacoustics) the lids of the operant box; one each on the Observer’s dry and Target’s wet side. USV data were recorded with a sampling rate of 250 kHz and analyzed with DeepSqueak version 2.6.0^57^ in MATLAB. Additional details are located in the supplemental methods. Calls were reviewed by researchers blind to experimental conditions. The proportion of calls that fell within the distress range (18–35 kHz) and prosocial range (< 35 kHz) compared to the rats’ total calls made per trial were calculated and compared on test days. In order to discern the impact the change in latency between baseline test day latencies (as calculated by the formula $Release Ratio = \frac{{Acq}_{Test}-{Acq}_{BL}}{{Acq}_{BL}}$; an increase in latency from BL to test days is reflected by a positive ratio) had on Targets’ affect, a correlation analysis was performed between the release ratio and the proportion of distress or prosocial calls made by Targets on those respective days.

### Experiment 1: Baclofen/muscimol inhibition of the AI

The AI of Observers (n = 8) were bilaterally cannulated for micro infusions. Rats were given one week to recover and then performed the targeted helping task for 8 days. On days 9–10, rats were randomly assigned to receive direct micro infusions of either phosphate-buffered saline (PBS), or a baclofen/muscimol (B/M) cocktail^58^ 30 min prior to the first trial of the day in a within-subjects design (Fig. 2A). Ultrasonic vocalizations (USVs) were recorded on test days as previously described to elucidate changes in affect caused by any alteration in helping behavior. Following behavior on day 10, Observers were sacrificed and placement of cannulae were grossly observed with the aid of a three-part manual quick stain (Mercedes Scientific, Lakewood Ranch, FL).

### Experiment 2: chemogenetic inhibition of the AI

Prior to experimentation, Observer rats were randomly assigned to receive micro injections of either the control viral construct, AAV8-CaMKIIα-enhanced green fluorescent protein (“EGFP,” Addgene; n = 8) or the inhibitory DREADD virus, AAV8-CaMKIIα-hM4D(Gi)-mCherry (“hM4Di,” Addgene; n = 10). Following viral infusion and incubation, rats performed targeted helping for 8 days. On test days 9–10, 30 min prior to behavioral testing, Observers received i.p. injections of either clozapine-N-oxide (CNO) hydrochloride (Hello Bio, Princeton, NJ), dissolved in water and administered at a dose of 10 mg/kg, or water (counter balanced) in a within-subjects design (Fig. 3A). Experimenters were blind to injection condition. Five days following targeted helping, rats underwent the social reward place conditioning task (Fig. 4A), in which animals also received CNO or water injections 30 min prior to days 2–4 of testing. Placement and DREADD efficacy were confirmed by double-labeling for the viral fluorophore and Fos + cell overlap within the AI (Fig. 3D).

### Data analysis

Latency to release the distressed conspecific was the primary dependent variable for the behavioral data. Acquisition was analyzed by a one-way repeated measures (RM) ANOVA across days for all experiments. In the B/M inhibition study (Experiment 1), a RM 2-way ANOVA was used to compare the change in chain pull latency between baseline acquisition (BL) and test days when rats received PBS or B/M infusions. Additionally, a 2-way ANOVA was performed to compare total vocalization call counts on test days. A mixed effects 2-way ANOVA was used to compare changes in distress vocalizations as a percent of total calls on test days, while a Pearson correlation was performed between Observers’ Test/BL latency ratio and their respective Targets’ proportion of distress calls. Finally, for the study examining the chemogenetic inhibition of the AI (Experiment 2), a RM 2-way ANOVA was used, with group (EGFP vs. hM4Di) and injection (water vs. CNO) as the two variables. One-way RM ANOVAs were also performed to elucidate the differences in time spent in the zones between groups during social reward place conditioning. Any post hoc comparisons were conducted using a Holm-Sidak’s correction for family wise error when appropriate, with the alpha set at 0.05. All analyses were conducted with Prism Software version 9, and data are expressed as the mean ± SEM.

## Supplementary Information

Supplementary Information.
