# Supplementary material for: The role of the anterior insula during targeted helping behavior in male rats
Source: Sci Rep. 2022 Feb 28;12:3315. doi: 10.1038/s41598-022-07365-3 (PMC8885669; doi:10.1038/s41598-022-07365-3)
Supplement: Supplementary file 1 — Supplementary Information. [file 41598_2022_7365_MOESM1_ESM.docx]

**Supplemental Methods**

Cannula Placement and Viral Infusions:

Experiment 2: Cannula Placement and Microinfusions: Observers (n=8) were bilaterally cannulated in the AI with two 26-gauge single barrel steel guide cannulae (Plastics One, Roanoke, VA), which were anchored to the skull with cranioplastic cement and steel screws (Plastics One). Rats underwent targeted helping as previously described for 8 days of acquisition. On days 9 and 10, awake Observers had either a baclofen/muscimol (B/M) cocktail (Tocris, Bristol, UK) dissolved in PBS, and combined in a 1.0mM/0.1 mM solution as previously described [41], or PBS (counter balanced) bilaterally microinfused at 0.5 μL/side into the AI [41] using a microinjector (Plastics One) that extended 1 mm beyond the implanted cannula via a gas-tight Hamilton syringe mounted on a microinfusion pump (Harvard Apparatus). The injectors were left in place for 5 min to allow for diffusion of the drug 30 minutes prior to behavior. Each animal served as its own control in a within-subjects model. Histological verification of microinjector needle placements within the anterior insula were collected and evaluated. Sections from representative animals are shown below.







Representative sample of cannula placement in the anterior insula. In all experiments, the AI was targeted using the following coordinates relative to the skull and bregma: +3 mm anteroposterior, ±4 mm mediolateral, and -4 mm (guide cannula) or -5mm (glass micropipette) dorsoventral according to a stereotaxic atlas [42].

Experiment 3: Viral Infusions: Observers were randomly selected to receive either AAV8-CaMKIIα-EGFP (n=8) or AAV8-CaMKIIα-hM4D(Gi)-mCherry (n=10). Viral vectors were directly infused into the AI of Observers using a glass micropipette via Auto-Nanoliter Injector (Nanoject II, Drummond Scientific) at the time of surgery. During intracranial surgery, the virus was infused at a volume of 50.6 nL/injection every 30 seconds at a rate of 23 nL/second followed by an additional 5 min. to allow the injected virus to diffuse prior to removal of the pipette. Cranial holes were covered with bone wax dental cement and the wound was sutured closed with 4–0 Perma-Hand silk sutures. DREADDs were allowed 3 weeks to reach maximal expression levels before behavioral testing began.

Social Reward Place Conditioning

On day 1, as a pre-interaction evaluation, rats were placed in the open field and given 10 min. to explore the environment. White tape was used to mark two 16 × 18 in. squares on the floor on opposite ends of the apparatus in order to indicate two zones; the social zone (SZ, the area with the unfamiliar rat) and the object zone (OZ, with a novel object) would go on subsequent sessions. The amount of time spent in each of these areas was recorded. On days 2, 3, and 4, an unfamiliar rat was placed in a wire cage (11 × 7 × 7 in) in the SZ, and a novel object (battery, pencil holder, or a soda can) was placed in the center of the OZ on the opposite end of the arena. Finally, on day 5, rats were returned to the empty open field for a post-interaction assessment. Each day, we evaluated the amount of time rats spent in the SZ and OZ throughout the 10 min. session.

Ultrasonic Vocalization Measurement and Analysis

Microphones were connected to Avisoft UltraSoundGate 416Hb multichannel recording system and processed using Avisoft-SASLab Pro software (Avisoft Bioacoustics, Glienicke, Germany). Microphones were placed above the Observer and the Target’s wet side of the chamber in order to best assess calls from both animals throughout the trial. USVs were recorded throughout the entirety (300s) of one trial during PBS and B/M test days (**Experiment 2**). Data were recorded with a sampling rate of 250kHz and analyzed with DeepSqueak version 2.6.0 [42] in MATLAB. Due to background noise during the task, post-hoc denoising was carried out and subsequently rechecked for errors by an experimenter. Calls with tonality of <0.35 were considered to be background and were rejected manually from analysis. Post-hoc denoising was carried out and subsequently reevaluated for errors by an experimenter. Remaining USVs were assigned to a particular animal by an experimenter blind to the conditions by comparing the start time and power of a call that was picked up by both microphones. Earlier start time and higher power indicated closer proximity to a particular microphone and was therefore assigned to the animal on that side of the chamber.

**Supplemental Figure 1**

**Supplemental Figure 1.** In **Experiment 2**, Observers had bilateral indwelling cannulae implanted into the anterior insula (AI) prior to testing. Following 8 days of acquisition, Observers either received direct microinfusions of baclofen/muscimol (B/M) or a phosphate buffered saline (PBS) control solution 30 minutes prior to targeted helping. **A.** Rats were tested in both conditions in a counter balanced order. On test days, ultrasonic vocalizations (USV) were recorded as described in **methods** and **supplemental methods**. Total call counts per session were analyzed with a mixed ANOVA with test session as within subjects and group as between subjects’ variables. No difference was seen between any group in the total number of calls made during test sessions. **B.** Total calls per session did not correlate with Release Ratio.

**Supplemental Figure 2.**

**Supplemental Figure 2**. In order to assess whether the changes seen during targeted helping following insula inhibition impacted other prosocial components, rats in **Experiment 3** underwent a social reward place conditioning task as described in **methods**. In addition to recording the latency Observers remained in either the social (SZ) or object (OZ) zones (**Figure 4**), the total duration the rats spent in nose-to-nose contact with the unfamiliar rat (**A**), as well as the duration spent climbing either the novel object (**B**) or the cage that housed the unfamiliar rat (**C**) was recorded. No difference was seen in any group. **D**) Additionally, total locomotion was recorded for all trials. A main effect of test days was found [F (2,26) = 91.9, p <0.0001], with post hoc analysis of the main effect indicating rats moved a greater distance on interaction days compared to pre-or post-interaction (p<0.0001). However, no effect of viral group was found.

|  | **Fos+** | **Virus+** | **Overlap** |
| --- | --- | --- | --- |
| **EGFP+H2O** | 147.5± 19.47 | 160.7±11.12 | \| 66.10±3.354 \| \| --- \| |
| **EGFP+CNO** | 116.9±6.559 | 149.5±7.858 | 70.12±4.475 |
| **hM4Di+H2O** | 110.7±5.520 | 151.2±8.103 | 67.21±1.314 |
| **hM4Di+CNO** | 69.2±2.853 | 182.0±8.899 | 35.27±3.206 |

**Supplemental Table 1**. CNO-mediated insula activity change in **Experiment 3** was determined by counting the overlap of Fos+ cells and either mCherry (hM4Di) or EGFP (control viral vector) under experimenter-blind conditions. Counts of Fos+, viral fluorophore+, and Fos+/virus+ overlap for each group are presented as mean ± SEM.
